# Skin changes in hairy cell leukemia

**DOI:** 10.1007/s00277-020-04349-z

**Published:** 2020-11-20

**Authors:** Ewa Robak, Dorota Jesionek-Kupnicka, Tadeusz Robak

**Affiliations:** 1 Department of Dermatology, Medical University of Lodz, Lodz, Poland; 2 Department of Pathology, Chair of Oncology, Medical University of Lodz, Lodz, Poland; 3 Department of Hematology, Medical University of Lodz, Lodz, Poland; 4 Copernicus Memorial Hospital, Ul. Ciolkowskiego 2, Lodz, Poland

**Keywords:** Adverse drug reactions, Cladribine, Interferon, Skin neoplasms, Infectious, Hairy cell leukemia, Leukemia cutis, Skin, Cutaneous, Neutrophilic dermatoses, Secondary cancer, Melanoma, Vasculitis, Vemurafenib

## Abstract

Skin lesions have been reported in about 10–12% of hairy cell leukemia (HCL) patients. Most are etiologically related to autoimmune or infectious processes, although secondary cutaneous neoplasms and drug-induced lesions are also reported. However, leukemia cutis with the direct infiltration of the skin by leukemic cells is extremely rare in HCL patients. This paper reviews the epidemiology, pathogenesis, clinical symptoms, diagnosis, and approach to treating skin lesions in HCL. A literature review of the MEDLINE database for articles in English concerning hairy cell leukemia, skin lesions, leukemia cutis, adverse events, infectious, cutaneous, drug reactions, neutrophilic dermatoses, secondary neoplasms, and vasculitis was conducted via PubMed. Publications from January 1980 to September 2020 were scrutinized. Additional relevant publications were obtained by reviewing the references from the chosen articles.

## Introduction

Hairy cell leukemia (HCL) is a rare form of B cell indolent lymphoid leukemia involving mature and post-germinal center B lymphocytes, generally affecting the bone marrow, peripheral blood, and spleen. The disease comprises 2–3% of all leukemias [1, 2]. There is a 4:1 male predominance, and Caucasians are more frequently affected than other ethnic groups. The annual incidence is between 2.9 and 4.7 per million people per year [3–5]. HCL is characterized by progressive pancytopenia, splenomegaly, and hypercellular bone marrow. Lymph node infiltration and involvement of extranodal organs are rather infrequent. Rare clinical manifestations of HCL are occasionally reported [6–8]. These include bulky abdominal lymphadenopathy; tumor masses in the mediastinum; paravertebral masses; pleural effusion and ascites; skin lesions; ocular-corneal involvement; uveitis; retinal artery thrombosis; neurological-meningeal compression; esophageal, gastric, and hepatic involvement; and skeletal lesion [1, 2]. Leukemic cells may also be identified in peripheral blood as mononuclear cells with abundant, slightly basophilic cytoplasm and circumferential cytoplasmic “hairy” projection. HCL cells typically show a distinctive immunophenotype coexpressing CD19, CD20, CD11c, CD25, CD103, and CD123. Recently, *BRAF*-V600E mutation has been discovered as specific oncogenic mutation for classic HCL. Another characteristic feature of classic HCL is the expression of annexin A1, which is easily detectable by immunohistochemical staining. Thanks to treatment with purine analogs, cladribine and pentostatin, the prognosis for classic HCL has improved from poor to highly treatable with near-normal survival [9, 10]. Purine analogs induce a durable and unmaintained remission in 76 to 98% of patients, with relapse rates of about 30 to 40% after 5 to 10 years of observation, and overall survival (OS) can be longer than 20 years in many cases [11, 12]. Moreover, in relapsed patients, subsequent complete remission (CR) can be achieved with re-treatment. However, treatment with purine analogs causes significant side effects, including infections and secondary malignancies related to profound and long-lasting immunosuppression [1]. Treatment with cladribine or pentostatin is not recommended for patients presenting with active infection and severe neutropenia when the risk of infection is high. Interferon-α (IFN-α) is rarely recommended front-line therapy for HCL because of its low CR rate. However, it may be still used in patients presenting with neutropenia below 0.2 × 109/L and during pregnancy [13, 14]. In the majority of patients, only partial responses (PR) can be achieved and prolonged treatment is necessary to maintain remission [15]. Another active drug in the treatment of HCL is rituximab. It can be given as a single agent or in combination with other agents, including purine nucleoside analogs [16]. The overall response (OR) rate ranges from 26 to 80% and CR rate from 13 to 53% of refractory or relapsing patients [17, 18]. Better results were observed when rituximab was combined with cladribine [19]. Recently, anti-CD22 immunotoxin moxetumomab pasudotox (Lumoxiti, Astra Zeneca) has been approved by the FDA for the treatment of relapsed or refractory HCL patients who have received at least two prior systemic therapies, including a purine nucleoside analog [20, 21]. The oral BRAF inhibitor vemurafenib (Zelboraf™, Roche) has been also shown to have antitumor activity in *BRAF* V600–mutated HCL [22]. It has been approved by the FDA for the therapy of *BRAF* V600E mutant metastatic melanoma. Vemurafenib exhibits a remarkable activity in multiple-relapsed and refractory HCL. Two other BRAF inhibitors, dabrafenib (Tafinlar®, GlaxoSmithKline) and encorafenib (LGX818, Novartis), are also potentially useful in the treatment of HCL [23]. Cutaneous symptoms have been reported in 10–12% of HCL patients [24]. While most of them were autoimmune or infectious in nature, secondary cutaneous neoplasms and drug-induced lesions have also been reported [25]. The direct infiltration of the skin (i.e., leukemia cutis) is extremely rare in HCL patients. However, cutaneous manifestations of HCL are not generally recognized as a diagnostic source and biopsies are seldom performed. This review examines the epidemiology, pathogenesis, clinical symptoms, diagnosis, and treatment of skin lesions in HCL.

## Leukemia cutis

Leukemia cutis is defined as infiltration of the epidermis, the dermis, and the subcutaneous tissue by leukemic cells [26, 27]. The most common types of leukemia cutis are observed in acute T cell leukemia/lymphoma (ATLL), chronic lymphocytic leukemia (CLL), and acute myeloid leukemia (AML) with monocytic or myelomonocytic subtypes, chronic lymphocytic leukemia (CLL), and T cell leukemia/lymphoma [27, 28]. Clinically, leukemia cutis manifests as maculopapular eruptions, nodules, infiltrative plaques, and ulcers. In most reported cases, biopsy has indicated that cell infiltrates are perivascular, involving the dermis and sparing the epidermis [27, 29–35]. Direct infiltration of the skin by leukemic cells is only occasionally observed in HCL. In addition, in most patients with HCL, the leukemic lesions were diagnosed only based on clinical examination without supporting histology [29]. Although the molecular mechanisms of the pathogenesis of leukemia cutis are not well defined, chemokine receptors and adhesion molecules may play important roles in the migration of leukemic cells into the skin via skin-selective homing processes [27]. CLA and CCR4 receptors on the circulating leukemic cells may interact with E-selectin and/or TARC/CCL17 on the dermal post-capillary venules; these may stimulate the rolling and tethering of leukemic cells into the dermis. The resulting interaction between integrins and endothelial-bound chemokines may stimulate the arrest of the leukemic cells and their transmigration into the dermis.

Clinical data from lager series and detailed single case reports have been published since 1980 [26, 29–35]. In a study of 48 patients reported as leukemia cutis among 600 HCL patients (8%), the condition was confirmed histologically in only eight (1.6%) [29]. Elsewhere, only one patient in a series of 113 patients (1.1%) with cutaneous findings had leukemic skin lesions; the other patients had non-specific cutaneous findings including recurrent infections, ecchymoses, petechiae, pallor, drug reactions and reactions to transfusions, and non-herpetic ulcerations [33]. While leukemia cutis has been diagnosed in the course of the disease in some patients, in most cases, it was observed at presentation of HCL [27, 29–36]. Seven cases of HCL with leukemia cutis that were described in detail in English language literature are summarized in Table 1. The diagnosis of leukemia cutis is based on a morphological pattern of skin infiltration, cytologic features, and the immunophenotype of the neoplastic cells [27]. Skin biopsy and immunophenotyping must be performed in all patients with suspicion of leukemia cutis. An established diagnosis of systemic leukemia is needed before a diagnosis of leukemia cutis can be confirmed. It is recommended that skin changes should be correlated with clinical features as well as with bone marrow and peripheral blood findings [27, 34].

**Table 1:** Characteristics of well-documented patents with hairy cell leukemia and specific skin involvement (leukemia cutis)

Authors	Age/gender	HCL status at skin lesion diagnosis	Clinical characteristic of skin lesions	Histology of skin lesions	Treatment of HCL and leukemia cutis
Lawrence et al. [31]	59/M	HCL in the skin, blood, and BM diagnosed at presentation	Multiple erythematous, slightly raised papules, and pustular lesions over all of the extremities, palms, and trunk	Throughout the dermis, large numbers of uniform, mononuclear cells were arranged in discrete patches, usually surrounding dermal blood vessels. Electron microscopy performed on the BM and skin biopsy specimens revealed cells consistent with a diagnosis of HCL	Skin lesions gradually disappeared over 1 to 2 weeks after splenectomy
Finan et al. [33]	74/M	HCL diagnosed 2 years before development of leukemia cutis	Bilateral violaceous infiltrative plaques over the temporal portion of the scalp and violaceous nodules and infiltrative plaques on the central part of the chest	Histology from punch biopsy specimens confirmed the diagnosis of leukemia cutis	Treatment with chlorambucil and prednisone ineffective; the patient died from infection
Arai et al. [29]	68/M	HCL in the skin, blood, and BM at presentation	Crops of 0.2–0.5-cm erythematous papules over the upper extremities	Light microscopy: tumor cells judged to be hairy cells spared the epidermis but infiltrated the upper dermis as patchy clusters around small blood vessels and skin; 80% of infiltrating hairy cells in the upper dermis showed diffuse cytoplasmic positivity by TRAP staining	IFN-α 3 million units per day for 16 weeks, when skin lesions had improved; retreated with IFN-α 6 million units per day—CR of skin lesions after 6 weeks
Bilsland et al. [30]	62/M	HCL in the skin, blood, and BM at presentation	Transient, widespread, non-pruritic skin eruption, with areas of erythema, purpura, and indurated plaques	Skin biopsy: infiltration of the papillary dermis by large mononuclear B cells surrounded by smaller lymphoid T cells	Eruption gradually disappeared 17 days after it first developed. Improvement after splenectomy and remission 45 months after presentation on IFN-α maintenance
Colovic et al. [26]	60/M	HCL in the skin and BM at presentation	Painless maculopapular, red brick skin infiltrates 1–2 cm in diameter of the almost whole skin	Perivascular and patchy infiltrates, composed of DBA44 positive small- to medium-sized lymphoid cells, with oval or indented nuclei, with homogenous, ground-glass chromatin, inconspicuous nucleoli, and abundant, pale blue cytoplasm	Cladribine (2 courses) and splenectomy, cutaneous lesion disappeared after HCL treatment
Ergene et al. [35]	59/M	HCL in the skin and BM at presentation	Pale skin tumor infiltrated the musculus pectoralis major with a diameter of 11 × 30 cm, with multiple scars attributed to sharp blade scars other than the main mass	Lymphoid cells with round nucleus and cytoplasm ridges in BM biopsy and epidermis-dermis, respectively. Flow cytometry—CD11c: 73.28%, CD19: 70.07%, CD22: 68.08%	Cladribine (1 course), complete response in BM, cutaneous infiltration completely disappeared, lasting remission at 5 years
Fino et al. [36]	47/M	HCL in the skin and BM at presentation	Purplish-brown skin nodule on the back of the left hand, 2 × 2 cm, with an erythematous halo, ulcerated surface, and squamous crusts	Histology: infiltration of dermal tissues by leukemoid cells positive for HCL surface markers and degenerative changes of the epidermis, dermoepidermal border, and collagen	Skin lesion was surgically removed

*BM* bone morrow, *CR* complete response, *HCL* hairy cell leukemia, *IFN-α* interferon-α, *TRAP* tartrate-resistant acid phosphatase

Leukemia cutis involvement in HCL has a variety of clinical appearances, including papules, plaques, or nodules ranging from violaceous to red-brown in color or flesh-colored nodules, occasionally with central ulceration. Skin lesions may be localized to one region or lesions can be generalized in many places. A differential diagnosis of leukemia cutis should include secondary cancers and inflammatory and infectious skin lesions [34]. Leukemia cutis in HCL patients responds well to antileukemic treatment with purine analogs. In most cases, cutaneous infiltrates disappeared with a complete resolution of the skin lesions when treatment with cladribine was used (Table 1) [27, 35].

## Secondary skin neoplasms

The association between HCL and second primary malignancies remains controversial [37–41]. The overall incidence of second primary tumors in patients with HCL ranges from 19.9 to 24% [41–45]. The most common include second primary malignancies of the skin, melanoma and non-melanoma skin cancer [43, 44]. Existing data suggests that patients with HCL have a higher risk of non-melanoma skin cancers [45]; therefore, these patients should be closely examined, especially when secondary risk factors exist like fair skin and extensive sun burden. In contrast, there is no clear evidence of increased melanoma cases in patients with HCL [45]. Recent data demonstrated a 10-year combined melanoma and non-melanoma skin cancer incidence of 11.3%, including 4.4% for melanoma and 6.9% for non-melanoma skin cancers [45]. In addition, data from Surveillance, Epidemiology and End Results (SEER) based on 4750 patients with HCL indicated a subsequent diagnosis of melanoma in 1.2%. However, standardized incidence ratios (SIRs) suggest that melanoma is not more common in HCL patients than in the general population. In addition, HCL patients diagnosed before the introduction of purine analog therapy in 1990 showed a similar incidence of melanoma to those diagnosed afterwards [45]. In contrast to these results other study showed higher rates of second primary malignancies since the introduction of purine analog treatment [41]. A recent study by Watts et al. reported no significantly increased risk of melanoma in HCL patients, compared to the general population [45].

Importantly, skin cancers in HCL patients have a high frequency of RAS mutations [46]. In a recent report, 33.3% of HCL patients with skin cancers had activating RAS mutations. Vemurafenib can further increase the incidence of secondary skin cancer events in melanoma patients [47, 48]. BRAF inhibition can accelerate the growth of cutaneous squamous cell carcinomas and melanoma resulting from the activation of MAPK signaling [49, 50]. However, the combination of a MEK inhibitor with a BRAF inhibitor can improve antileukemic efficacy and reduce the frequency of secondary cutaneous malignancies in melanoma and HCL patients [51–55].

The development of cutaneous T cell lymphoma and HCL is a very rare phenomenon. We found six cases of concurrent mycosis fungoides and HCL in the English language literature [56–59]. Another report describes a case of reactive polyclonal T cell lymphocytosis mimicking Sezary syndrome in an HCL patient [60]. The simultaneous diagnosis of a primary cutaneous form of peripheral T cell lymphoma (PTCL) and a variant of HCL has been also reported [56].

Concurrent metastatic Merkel cell carcinoma (MCC) and HCL have also been observed at initial presentation [61]. The authors describe a patient with a markedly swollen left leg, with several small skin nodules and similar lesions on the right upper back. Bone marrow biopsy revealed concurrent HCL and MCC. The biopsy from the skin lesion also confirmed MCC.

## Vasculitis syndromes

**Fig. 1:**
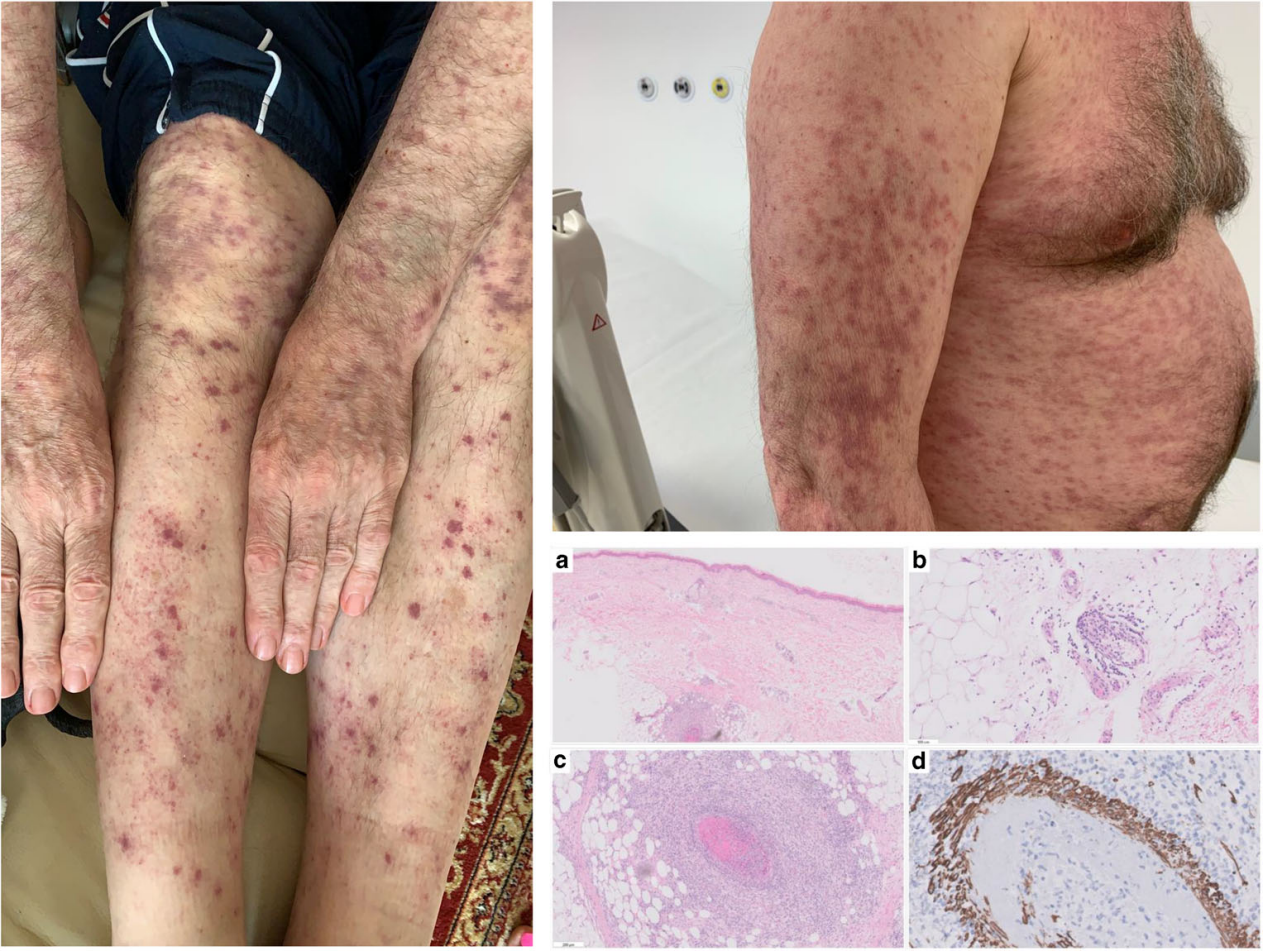
Maculopapular skin rash due to paraneoplastic dermatitis with cutaneous leukocytoclastic vasculitis on upper and lower extremities at diagnosis of HCL. Similar lesions were present on the chest, back, and neck. Skin biopsy with granulocytic and lymphocytic inflammation in small and large vessels in subcutaneous tissue (**a**, **b**, **c**) (HE × 20, × 100, × 200); fibrinoid necrosis and leukocytoclasis (fragmented neutrophilic nuclei) (**c**) with destruction of vessel wall (immunohistochemical staining for desmin, DAKO, × 200) (**d**)

Vasculitis syndromes including cutaneous leukocytoclastic vasculitis (LCV), polyarteritis nodosa, and paraneoplastic vasculitis may predate the diagnosis of the lymphoid malignancies, including HCL [62, 63]. Vasculitis has been reported in 4.5–8% of cases with lymphoid malignancies and has occasionally been described in patients with HCL [63, 64]. In HCL patients, vasculitis may occur as a reaction to infection or to leukemia itself as a paraneoplastic syndrome (Fig. 1) [65–68].

A retrospective analysis of 129 patients with HCL between 1976 and 1983 identified two cases with symptoms of systemic vasculitis [68]. Another study of 42 HCL patients with coexisting vasculitis identified 21 patients with cutaneous leukocytoclastic vasculitis (CLCV) and 17 cases with panarteritis nodosa (PAN) [69]. PAN generally occurred after HCL and splenectomy and was often preceded by infection. Vasculitis with skin erythema can be an initial manifestation of early-stage HCL [67, 70]; it can also precede the clinical manifestations of HCL or follow a diagnosis of HCL (Fig. 1) [67, 69, 71, 72]. Although the pathogenesis of vasculitis is unknown, it is believed that immune complex injury and cross-reactions between autoantibodies to hairy cells and endothelial cells may take part [68, 69, 73]. In some patients, perivascular accumulation of leukemic cells among the cells of the perivascular infiltrations in the skin biopsy can occur [70]. In most cases, resolution of symptoms was observed after specific antileukemic treatment, and HCL is considered an underlying etiology for leukocytoclastic vasculitis, particularly in newly diagnosed patients [72]. Several reports indicate that HCL patients with coexisting vasculitis respond well to corticosteroids, splenectomy, and cytotoxic therapy with IFN-α or purine analogs [74]. In particular, treatment with cladribine improves the cutaneous symptoms and complete regression of vasculitis was usually achieved [61, 67, 69, 70, 74]. On the other hand, cladribine itself can induce vasculitis in HCL patients [75, 76].

Behçet’s disease is a multisystemic vasculitis involving both arteries and veins, which is characterized by recurrent oral and genital ulcers with characteristic cutaneous and ocular features recognized by a positive pathergy test [77]. This disease is observed in various hematological malignancies, including leukemias. However, it is often difficult to establish whether Behçet’s disease is associated with leukemia, or it is merely a coexisting, leukemia-independent disease. Only two cases of HCL being associated with Behçet’s disease have been reported in the literature: the first described a patient with HCL who developed Behçet’s disease [78], and the second described the initial presentation as arthritis, oral and genital ulcerations, and papulopustular skin lesions in addition to pancytopenia [79].

Polyarteritis nodosa is an immune complex-mediated necrotizing vasculitis of small- and medium-sized arteries typically with multiorgan involvement. It is characterized by segmental transmural inflammation of muscular arteries. Patients may present with cutaneous involvement, especially livedo reticularis, painful peripheral neuropathy, musculoskeletal pain, and vascular nephropathy. Several investigators have indicated an association between HCL and PAN, and an etiologic relationship between the two conditions has been suggested [69, 80–82]. The pathogenetic mechanism of PAN in HCL includes hepatitis B antigenemia, direct invasion of blood vessel wall by leukemic cells, cross-reactivity of antibodies that target surface determinants on hairy cells with epitopes on endothelial cells, and splenectomy [68, 83, 84].

## Neutrophilic dermatoses

Neutrophilic dermatoses include Sweet’s syndrome, pyoderma gangrenosum and neutrophilic eccrine hidradenitis [85]. Sweet’s syndrome, also known as acute febrile neutrophilic dermatosis, is a rare inflammatory condition that can be associated with drugs, infections, inflammatory bowel disease, pregnancy, and cancer among others. It typically presents with acute onset dermal neutrophilic lesions, leukocytosis, and fever. The skin symptoms are characterized by erythematous painful lesions on the skin, which are distinctive, asymmetric, erythematous, and often tender plaques. The pathogenetic mechanism of the disease is not completely defined, but it may occur as a hypersensitivity reaction to an infection, cancer, or other diseases. Sweet’s syndrome has been rarely reported in association with HCL [86, 87]. A chemoattractant substance released from leukemic cells, including IL-8, and IFN-γ and granulocyte colony-stimulating factor (G-CSF) may be involved in developing neutrophilic tissue infiltration. Several studies have suggested a link between HCL and Sweet’s syndrome [88–94]. It can be the first manifestation of HCL, or diagnosed at relapse of leukemia. In the reported cases, the skin symptoms regressed after splenectomy or treatment with IFN**-**α [92, 93]. However, treatment with cladribine and prednisone seems to be the treatment of choice in HCL-associated Sweet’s syndrome [86, 89, 91, 93].

**Fig. 2:**
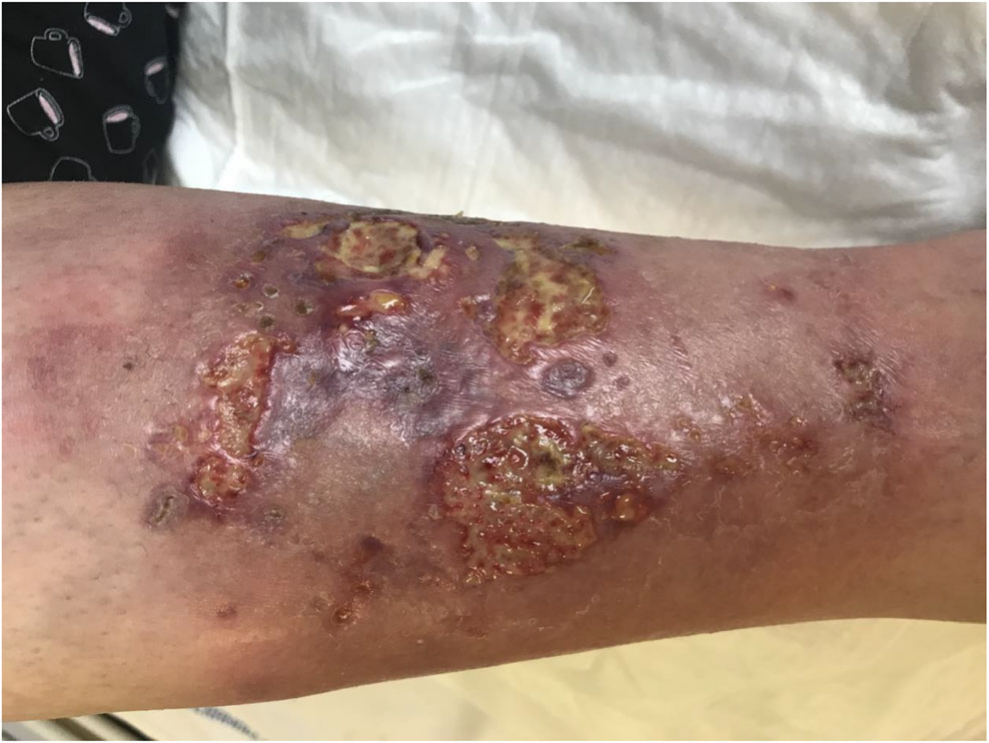
Ulcerative pyoderma gangrenosum on the right tibial surface in the patient with hairy cell leukemia before treatment. Lesions completely resolved after treatment with cladribine treatment

Pyoderma gangrenosum is a rare painful neutrophilic, reactive, non-infectious, inflammatory dermatosis involving the skin, mucosal areas, and other organs. Pyoderma gangrenosum is seen mostly in association with systemic diseases like hematologic disorders, inflammatory diseases, and arthritis [94]. The disease is manifested as painful erythematous lesion rapidly progressing to a blistered or necrotic ulcer (Fig. 2). Pyoderma gangrenosum exists as ulcerative, vegetative, pustular, bullous, and peristomal variants [95]. Although its pathogenesis is not fully understood, immune dysregulation involving neutrophil chemotaxis has been suggested as an important pathogenic factor. The disease can be associated with systemic disorders including hematologic malignancies. Pyoderma gangrenosum is rarely reported in HCL, and only few cases have been reported so far [96–99]; however, in such cases, the condition can be treated successfully with cladribine, without any additional use of immunosuppressive drugs such as corticosteroids or cyclosporine [97, 98]. The high efficacy of cladribine is believed to result from its antileukemic and immunosuppressive properties.

## Cutaneous infections

**Fig. 3:**
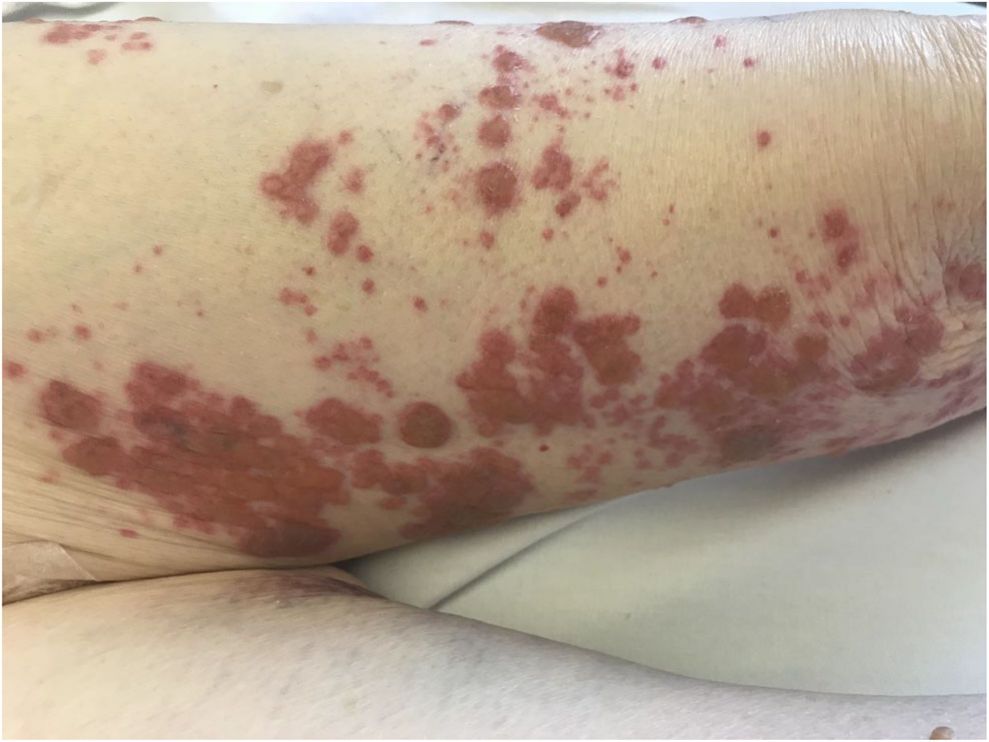
Herpes zoster infection in a patient with hairy cell leukemia treated with cladribine

Approximately 30% of HCL cases present as an infectious episode. Infections are also the most common cause of morbidity and mortality in HCL patients. These are most often bacterial or viral, especially Zoster infections (Fig. 3). In a study of 113 patients, 62 demonstrated both cutaneous and extra cutaneous infections (55%) [100]. Herpetic lesions were the most common, observed in 25 patients, including herpes simplex in 17 patients, Zoster in seven, and generalized varicella in one patient. Verrucae, dermatophyte, candidal infections, and pyogenic infections (i.e., abscesses, cellulitis, folliculitis, and pyoderma) were less frequently observed.

Opportunistic skin infections, including atypical mycobacterial skin infections, have also been reported in HCL patients [101, 102]. Trizna et al. present the case of a patient with HCL, who developed cutaneous tumor caused by *Mycobacterium kansasii*: surgical removal of the tumor and subsequent combination antituberculotic treatment with IFN-α led to resolution of infection and remission of HCL [103]. Another report described the successful ethambutol, cycloserine, and isoniazid treatment of a patient with HCL and pulmonary infection diagnosed with disseminated cutaneous *Mycobacterium malmoense* infection [104]. Maurice et al. describe the case of a 66-year-old man with HCL treated successfully with IFN-α, who developed widespread cutaneous and subcutaneous nodules caused by intracellular *Mycobacterium avium* infection [105]. The skin lesions progressed slowly on quadruple antituberculous chemotherapy; however, a marked improvement of all skin lesions was observed on erythromycin therapy.

Rare cases of opportunistic fungal infection were also described in HCL patients. Kumar et al. reported a case of cutaneous *Sporotrichosis* infection as a presenting manifestation of HCL. The lesions resolved following antifungal therapy [106]. Opportunistic infection due to *Listeria monocytogenes* manifesting as cerebritis and cutaneous lesions was also reported in a patient with HCL [107].

**Fig. 4:**
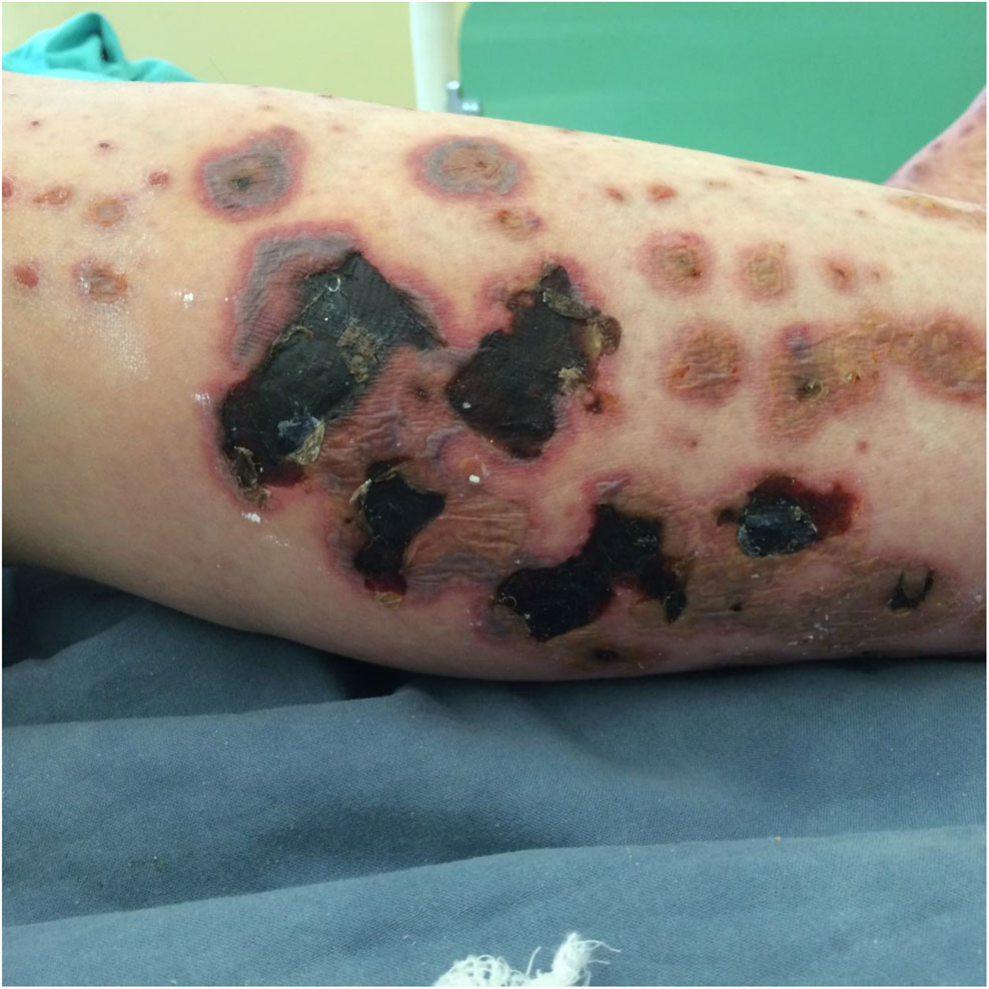
Ecthyma gangrenosum in the patient with hairy cell leukemia associated with *Pseudomonas aeruginosa* bacteremia, manifested as painless red macule with surrounding erythema, followed by hemorrhagic bullae and cutaneous ulcerative lesions

Ecthyma gangrenosum is a rare cutaneous ulcerative lesion associated with *Pseudomonas aeruginosa* infection, but is also observed in patients with other bacterial, viral, and fungal infections. Typically it starts as a painless red macule that rapidly becomes pustular with surrounding erythema, followed by hemorrhagic bullae and cutaneous ulcerative lesions (Fig. 4) [108]. Ecthyma gangrenosum has been rarely observed in HCL patients [109]. As ecthyma gangrenosum is typically associated with *Pseudomonas aeruginosa* bacteremia, the diagnosis should be followed by immediate empiric antimicrobial therapy with an antipseudomonal antibiotic [109]. However, in HCL patients, simultaneous treatment with purine analogs can be more effective. A recent study reported no clinical improvement in a patient simultaneously diagnosed with HCL and ecthyma gangrenosum, despite adequate antibiotic treatment with ceftazidime, clindamycin, and gentamicin [109]; however, the ecthyma gangrenosum resolved completely within 3 months after treatment with cladribine, and CR was achieved for HCL.

## Cutaneous adverse drug reactions

A high incidence of cutaneous adverse drug reactions has been reported in patients with hairy cell leukemia treated with cladribine [66, 110–112]. Although cladribine has been reported to cause rash during therapy, any adverse skin reactions are unlikely to be directly attributable to cladribine itself in most patients, but rather to other concomitantly used drugs, particularly antibiotics. It is suspected that cladribine predisposes patients with HCL to hypersensitivity to concomitant medications, most commonly allopurinol, penicillins, trimethoprim-sulfamethoxazole, or granulocyte-colony stimulating factor [110, 113, 114].

Recently, Castagna et al. described 12 HCL patients treated with cladribine who developed cutaneous adverse drug reactions during the aplasia stage, with a median occurrence at 12 days (10–15) after cladribine initiation [115]. Eight patients had maculopapular exanthema with systemic symptoms, two patients had acute generalized exanthematous pustulosis, and another two had DRESS (drug rash with eosinophilia and systemic symptoms) syndrome. Eleven patients were demonstrated drug allergies.

Meher-Homji et al. compared the prevalence of antibiotic allergy labels (AAL) in 43 cladribine-treated HCL patients, with those in patients with hematological malignancies not treated with cladribine and those in patients with CLL or follicular lymphoma treated with fludarabine [111]. A higher prevalence of antibiotic allergy was observed in cladribine-treated patients with HCL (26/43 patients, 60.47%), than in non-HCL cladribine-treated controls (14%) and controls treated with fludarabine (25%). Among the 26 antibiotic-allergic patients, only five had undergone allergological investigations with skin tests, four of whom were found to be positive for antibiotics. Another study reported that the development of halogenoderma with skin necrosis over the forearm, requiring excision and skin grafting, was also reported in a patient with HCL treated with intravenous cladribine [116].

IFN**-**α treatment is associated with the incidence of common rashes in 2% of patients, as indicated by the drug manufacturer. IFN**-**α can also induce alopecia universalis and cutaneous polyarteritis nodosa. Localized reactions include hyperpigmentation of the tongue and facial erythema. Severe polymorphic erythema and blisters have been also reported after the application of IFN-α to treat HCL. In this case, the patient developed a rash and erythema without pruritus and fever after the first injection of IFN-α, a rash on the neck after the second injection, and diffuse erythema, rashes, and blisters after the third injection. Histopathological examination of a skin biopsy showed lymphocytic exocytosis and a perivascular lymphocytic infiltration. The skin symptoms receded after treatment with methylprednisolone [117].

Another study describes the occurrence of disseminated ulcerating lupus panniculitis during IFN-α therapy of HCL [118]. The patient initially responded to oral prednisone and hydroxychloroquine became resistant to the treatment after several months. However, the skin lesions were controlled by treatment with cladribine and rituximab. The responsiveness of skin disease and HCL to cladribine and rituximab suggests a common denominator in pathogenesis of both disorders.

The adverse effects of vemurafenib are mostly associated with the skin. These include Grover’s disease, photosensitivity, rash, palmar fibrosis, and warts. Vemurafenib was also associated with other hyperkeratotic cutaneous adverse reactions including squamous cell carcinoma, plantar hyperkeratosis, verrucal keratosis, and keratosis pilaris–like reactions [119, 120]. Grover’s disease is an acquired skin disorder characterized by pruritic papulovesicular eruptions, edematous papules, and/or papulovesicles of the trunk with acantholysis of the epidermis in histopathological examination [121]. Grover’s disease was observed in 42.9% of metastatic melanoma patients treated with a single BRAF inhibitor [119]. However, Grover’s disease has also been observed in HCL patients not treated with BRAF inhibitors [122].

## Conclusions

Specific lesions occur in approximately 10–12% of HCL patients. Cutaneous manifestations are varied and are mostly related to the occurrence of infection, autoimmune processes, secondary neoplasms, medication use, or specific leukemic infiltration. Vasculitic manifestations are relatively uncommon events in HCL patients and can occur anytime during the course of HCL, sometimes preceding the clinical manifestations of leukemia. Many vasculitides have been associated with HCL, including polyarteritis nodosa, leukocytoclastic vasculitis, and pyoderma gangrenosum. Treatment of the underlying HCL, especially with purine analogs, may generally result in a rapid improvement of the cutaneous lesions and potentially complete regression of the vasculitis. The direct leukemic infiltration of the skin (i.e., leukemia cutis) is a relatively rare manifestation of the disease; however, a response to the specific treatment for HCL resulted in the disappearance of cutaneous infiltrates in most presented cases. The HCL population seems to be more susceptible to the incidence of secondary cutaneous malignancies, particular melanoma, than the general population. In addition, skin cancers have a high frequency of RAS mutations in HCL patients and may behave unusually aggressively. A high incidence of cutaneous adverse drug reactions, especially in treated patients with HCL, was observed, particularly in patients receiving cladribine and concomitant medications. Finally, the use of antileukemic drugs in HCL predisposes the patient to viral, fungal, and bacterial skin infections, including opportunistic infections.

## References

[1] Grever MR, Abdel-Wahab O, Andritsos LA, Banerji V, Barrientos J, Blachly JS, Call TG, Catovsky D, Dearden C, Demeter J, Else M, Forconi F, Gozzetti A, Ho AD, Johnston JB, Jones J, Juliusson G, Kraut E, Kreitman RJ, Larratt L, Lauria F, Lozanski G, Montserrat E, Parikh SA, Park JH, Polliack A, Quest GR, Rai KR, Ravandi F, Robak T, Saven A, Seymour JF, Tadmor T, Tallman MS, Tam C, Tiacci E, Troussard X, Zent CS, Zenz T, Zinzani PL, Falini B (2017). Consensus guidelines for the diagnosis and management of patients with classic hairy cell leukemia. Blood.

[2] Robak T, Matutes E, Catovsky D, Zinzani PL, Buske C, ESMO Guidelines Committee (2015). Hairy cell leukaemia: ESMO Clinical practice guidelines for diagnosis, treatment and follow-up. Ann Oncol.

[3] Teras LR, Desantis DE, Cerhan JR, Morton LM, Jemal A, Flowers CR (2016). 2016 US lymphoid malignancy statistics by World Health Organization subtypes. CA Cancer J Clin.

[4] Bernstein L, Newton P, Ross RK (1990). Epidemiology of hairy cell leukemia in Los Angeles County. Cancer Res.

[5] Kristinsson SY, Vidarsson B, Agnarsson BA, Haraldsdottir V, Olafsson O, Johannesson GM, Eyjolfsson GI, Bjornsdottir J, Onundarson PT, Reykdal S (2002). Epidemiology of hairy cell leukemia in Iceland. Hematol J.

[6] Polliack A (2002). Hairy cell leukemia: biology, clinical diagnosis, unusual manifestations and associated disorders. Rev Clin Exp Hematol.

[7] Tadmor T, Polliack A (2011). Unusual clinical manifestations, rare sites of involvement, and the association of other disorders with hairy cell leukemia. Leuk Lymphoma.

[8] Robak P, Jesionek-Kupnicka D, Kupnicki P, Polliack A, Robak T (2020). Bone lesions in hairy cell leukemia: diagnosis and treatment. Eur J Haematol.

[9] Piro LD, Carrera CJ, Carson DA, Beutler E (1990). Lasting remissions in hairy cell leukemia induced by a single infusion of 2-chlorodeoxyadenosine. N Engl J Med.

[10] Chandran R, Gardiner SK, Smith SD, Spurgeon SE (2013). Improved survival in hairy cell leukaemia over three decades: a SEER database analysis of prognostic factors. Br J Haematol.

[11] Else M, Dearden CE, Matutes E, Garcia-Talavera J, Rohatiner AZ, Johnson SA, O'Connor NT, Haynes A, Osuji N, Forconi F, Lauria F, Catovsky D (2009). Long-term follow-up of 233 patients with hairy cell leukaemia treated initially with pentostatin or cladribine at a median of 16 years. Br J Haematol.

[12] Sigal DS, Sharpe R, Burian C, Saven A (2010). Very long-term eradication of minimal residual disease in patients with hairy cell leukemia after a single course of cladribine. Blood.

[13] Baer MR, Ozer H, Foon KA (1992). Interferon-alpha therapy during pregnancy in chronic myelogenous leukaemia and hairy cell leukaemia. Br J Haematol.

[14] Habermann TM, Rai K (2011). Historical treatments of in hairy cell leukemia, splenectomy and interferon: past and current uses. Leuk Lymphoma.

[15] Seymour JF, Estey EH, Keating MJ, Kurzrock R (1995). (1995) Response to interferon-α in patients with hairy cell leukemia relapsing after treatment with 2-chlorodeoxyadenosine. Leukemia.

[16] Leclerc M, Suarez F, Noël MP, Vekhoff A, Troussard X, Claisse JF, Thieblemont C, Maloisel F, Beguin Y, Tamburini J, Barbe C, Delmer A (2015). Rituximab therapy for hairy cell leukemia: a retrospective study of 41 cases. Ann Hematol.

[17] Thomas DA, O’Brien S, Bueso-Ramos C, Faderl S, Keating MJ, Giles FJ, Cortes J, Kantarjian HM (2003). Rituximab in relapsed or refractory hairy cell leukemia. Blood.

[18] Nieva J, Bethel K, Saven A (2003). Phase 2 study of rituximab in the treatment of cladribine-failed patients with hairy cell leukemia. Blood.

[19] Chihara D, Kantarjian H, O'Brien S, Jorgensen J, Pierce S, Ferrajoli A, Poku R, Jain P, Thompson P, Brandt M, Luthra R, Burger J, Keating M, Ravandi F (2016). Long-term durable remission by cladribine followed by rituximab in patients with hairy cell leukaemia: update of a phase II trial. Br J Haematol.

[20] Robak T, Wolska A, Robak P (2015). Potential breakthroughs with investigational drugs for hairy cell leukemia. Expert Opin Investig Drugs.

[21] Kreitman RJ, Dearden C, Zinzani PL, Delgado J, Karlin L, Robak T, Gladstone DE, le Coutre P, Dietrich S, Gotic M, Larratt L, Offner F, Schiller G, Swords R, Bacon L, Bocchia M, Bouabdallah K, Breems DA, Cortelezzi A, Dinner S, Doubek M, Gjertsen BT, Gobbi M, Hellmann A, Lepretre S, Maloisel F, Ravandi F, Rousselot P, Rummel M, Siddiqi T, Tadmor T, Troussard X, Yi CA, Saglio G, Roboz GJ, Balic K, Standifer N, He P, Marshall S, Wilson W, Pastan I, Yao NS, Giles F (2018). Moxetumomab pasudotox in relapsed/ refractory hairy cell leukemia. Leukemia.

[22] Tiacci E, Park JH, De Carolis L, Chung SS, Broccoli A, Scott S, Zaja F, Devlin S, Pulsoni A, Chung YR, Cimminiello M, Kim E, Rossi D, Stone RM, Motta G, Saven A, Varettoni M, Altman JK, Anastasia A, Grever MR, Ambrosetti A, Rai KR, Fraticelli V, Lacouture ME, Carella AM, Levine RL, Leoni P, Rambaldi A, Falzetti F, Ascani S, Capponi M, Martelli MP, Park CY, Pileri SA, Rosen N, Foà R, Berger MF, Zinzani PL, Abdel-Wahab O, Falini B, Tallman MS (2015). Targeting mutant BRAF in relapsed or refractory hairy-cell leukemia. N Engl J Med.

[23] Vergote V, Dierickx D, Janssens A, Verhoef G, Tousseyn T, Vandenberghe P, Wolter P, Delforge M (2014). Rapid and complete hematological response of refractory hairy cell leukemia to the BRAF inhibitor dabrafenib. Ann Hematol.

[24] Cannon T, Mobarek D, Wegge J, Tabbara IA (2008). Hairy cell leukemia: current concepts. Cancer Investig.

[25] Carsuzaa F, Pierre C, Jaubert D, Viala JJ (1994). Cutaneous findings in hairy cell leukemia. Review of 84 cases. Nouv Rev Fr Hematol.

[26] Colović N, Perunicić M, Jurisić V, Colović M (2010). Specific skin lesions in hairy cell leukemia at presentation: case report and review of literature. Med Oncol.

[27] Cho-Vega JH, Medeiros LJ, Prieto VG, Vega F (2008). Leukemia cutis. Am J Clin Pathol.

[28] Robak E, Robak T (2007). Skin lesions in chronic lymphocytic leukemia. Leuk Lymphoma.

[29] Arai E, Ikeda S, Itoh S, Katayama I (1988). Specific skin lesions as the presenting symptom of hairy cell leukemia. Am J Clin Pathol.

[30] Bilsland D, Shahriari S, Douglas WS, Chaudhuri AK, Todd WT (1991). Transient leukaemia cutis in hairy-cell leukaemia. Clin Exp Dermatol.

[31] Lawrence DM, Sun NCJ, Mena R, Moss R (1983). Cutaneous lesions in hairy cell leukemia. Case report and review of the literature. Arch Dermatol.

[32] Fressoldati A, Lamparelli T, Federico M, Annino L, Capnist G, Pagnucco G, Dini E, Resegotti L, Damasio EE, Silingardi V (1994). Hairy cell leukemia: a clinical review based on 725 cases of the Italian Cooperative Group (ICGHCL). Leuk Lymphoma.

[33] Finan MC, Su WP, Li CY (1998). Cutaneous findings in hairy cell leukemia. J Am Acad Dermatol.

[34] Parsi M, Go MS, Ahmed A (2020) Cancer, leukemia cutis. 2020 Aug 8. In: StatPearls [Internet]. Treasure Island: StatPearls Publishing; 2020 Jan.

[35] Ergene U, Ozbalcı D, Işisağ A (2012). Hairy cell leukemia and cutaneous involvement. Transfus Apher Sci.

[36] Fino P, Fioramonti P, Onesti MG, Passaretti D, Scuderi N (2012). Skin metastasis in patient with hairy cell leukemia: case report and review of literature. In Vivo.

[37] Dearden CE, Else M, Catovsky D (2011). Long-term results for pentostatin and cladribine treatment of hairy cell leukemia. Leuk Lymphoma.

[38] Au WY, Klasa RJ, Gallagher R, Le N, Gascoyne RD, Connors JM (1998). Second malignancies in patients with hairy cell leukemia in British Columbia: a 20-year experience. Blood.

[39] Saven A, Burian C, Koziol JA, Piro LD (1992). Long-term follow-up of patients with hairy cell leukemia after cladribine treatment. Blood.

[40] Kurzrock R, Strom SS, Estey E, O’Brien S, Keating MJ, Jiang H, Adams T, Talpaz M (1997). Second cancer risk in hairy cell leukemia: analysis of 350 patients. J Clin Oncol.

[41] Hisada M, Chen BE, Jaffe ES, Travis LB (2007). Second cancer incidence and cause-specific mortality among 3104 patients with hairy cell leukemia: a population-based study. J Natl Cancer Inst.

[42] Benz R, Arn K, Andres M, Pabst T, Baumann M, Novak U, Hitz F, Hess U, Zenhaeusern R, Chalandon Y, Mey U, Blum S, Rauch D, O’Meara Stern A, Cantoni N, Bargetzi M, Bianchi-Papina E, Rossi D, Passweg J, Lohri A, Berardi S, Li Q, Feller A, Stussi G (2020). Prospective long-term follow-up after first-line subcutaneous cladribine in hairy cell leukemia: a SAKK trial. Blood Adv.

[43] da Silva WF, Neto AC, da Rosa LI, de Siqueira IA, Amarante GD, Velloso EDRP, Rego EM, Rocha V, Buccheri V (2019). Outcomes and second neoplasms in hairy cell leukemia: a retrospective cohort. Leuk Res.

[44] Schön MP, Reifenberger J, Von Schmiedeberg S, Megahed M, Lang K, Gattermann N, Meckenstock G, Goerz G, Ruzicka T (1999). Multiple basal cell carcinomas associated with hairy cell leukaemia. Br J Dermatol.

[45] Watts JM, Kishtagari A, Hsu M, Lacouture ME, Postow MA, Park JH, Stein EM, Teruya-Feldstein J, Abdel-Wahab O, Devlin SM, Tallman MS (2015). Melanoma and non-melanoma skin cancers in hairy cell leukaemia: a Surveillance, Epidemiology and End Results population analysis and the 30-year experience at Memorial Sloan Kettering Cancer Center. Br J Haematol.

[46] Su F, Viros A, Milagre C, Trunzer K, Bollag G, Spleiss O, Reis-Filho JS, Kong X, Koya RC, Flaherty KT, Chapman PB, Kim MJ, Hayward R, Martin M, Yang H, Wang Q, Hilton H, Hang JS, Noe J, Lambros M, Geyer F, Dhomen N, Niculescu-Duvaz I, Zambon A, Niculescu-Duvaz D, Preece N, Robert L, Otte NJ, Mok S, Kee D, Ma Y, Zhang C, Habets G, Burton EA, Wong B, Nguyen H, Kockx M, Andries L, Lestini B, Nolop KB, Lee RJ, Joe AK, Troy JL, Gonzalez R, Hutson TE, Puzanov I, Chmielowski B, Springer CJ, McArthur GA, Sosman JA, Lo RS, Ribas A, Marais R RAS mutations in cutaneous squamous-cell carcinomas in patients treated with BRAF inhibitors. N Engl J Med 366(3):207–215 10.1056/NEJMoa1105358 PMC3724537 22256804

[47] Lacouture ME, Duvic M, Hauschild A, Prieto VG, Robert C, Schadendorf D, Kim CC, McCormack CJ, Myskowski PL, Spleiss O, Trunzer K, Su F, Nelson B, Nolop KB, Grippo JF, Lee RJ, Klimek MJ, Troy JL, Joe AK (2013). Analysis of dermatologic events in vemurafenib-treated patients with melanoma. Oncologist.

[48] Zimmer L, Hillen U, Livingstone E, Lacouture ME, Busam K, Carvajal RD, Egberts F, Hauschild A, Kashani-Sabet M, Goldinger SM, Dummer R, Long GV, McArthur G, Scherag A, Sucker A, Schadendorf D (2012). Atypical melanocytic proliferations and new primary melanomas in patients with advanced melanoma undergoing selective BRAF inhibition. J Clin Oncol.

[49] Robert C, Arnault JP, Mateus C (2011). RAF inhibition and induction of cutaneous squamous cell carcinoma. Curr Opin Oncol.

[50] Oberholzer PA, Kee D, Dziunycz P, Sucker A, Kamsukom N, Jones R, Roden C, Chalk CJ, Ardlie K, Palescandolo E, Piris A, MacConaill LE, Robert C, Hofbauer GF, McArthur GA, Schadendorf D, Garraway LA (2012). RAS mutations are associated with the development of cutaneous squamous cell tumors in patients treated with RAF inhibitors. J Clin Oncol.

[51] Flaherty KT, Infante JR, Daud A, Gonzalez R, Kefford RF, Sosman J, Hamid O, Schuchter L, Cebon J, Ibrahim N, Kudchadkar R, Burris HA, Falchook G, Algazi A, Lewis K, Long GV, Puzanov I, Lebowitz P, Singh A, Little S, Sun P, Allred A, Ouellet D, Kim KB, Patel K, Weber J (2012). Combined BRAF and MEK inhibition in melanoma with BRAF V600 mutations. N Engl J Med.

[52] Kreitman RJ, Moreau P, Hutchings M, Gazzah A, Blayn JY, Wainberg ZA, Stein A, Dietrich S, de Jonge MJA, Willenbacher W, De Greve J, Arons E, Ravandi F, Rangwala F, Burgess P, Mookerjee B, Subbiah V (2018) Treatment with combination of dabrafenib and trametinib in patients with recurrent/refractory BRAF V600E-mutated hairy cell leukemia (HCL). Blood 132 Abstract 391

[53] Caeser R, Collord G, Yao WQ, Chen Z, Vassiliou GS, Beer PA, Du MQ, Scott MA, Follows GA, Hodson DJ (2019). Targeting MEK in vemurafenib resistant hairy cell leukemia. Leukemia..

[54] Bhangoo MS, Saven A (2019). Secondary malignancies after treatment with single-agent vemurafenib in two patients with refractory hairy cell leukemia. Leuk Lymphoma.

[55] Zheng G, Chattopadhyay S, Sud A, Sundquist K, Sundquist J, Försti A, Houlston R, Hemminki A, Hemminki K (2019). Second primary cancers in patients with acute lymphoblastic, chronic lymphocytic and hairy cell leukaemia. Br J Haematol.

[56] Paolini R, Poletti A, Ramazzina E, Menin C, Santacatterina M, Montagna M, Bonaldi L, Del Mistro A, Zamboni S, D’Andrea E (2000). Co-existence of cutaneous T-cell lymphoma and B hairy cell leukemia. Am J Hematol.

[57] Hallermann C, Kaune MK, Tiemann M, Kunze E, Griesinger F, Mitteldorf C, Bertsch HP, Neumann C (2007). High frequency of primary cutaneous lymphomas associated with lymphoproliferative disorders of different lineage. Ann Hematol.

[58] Wong E, Mahmood MN, Salopek TG (2017). Concomitant B hairy cell leukemia and mycosis fungoides in an elderly man. Case Rep Dermatol.

[59] Barzilai A, Trau H, David M, Feinmesser M, Bergman R, Shpiro D, Schiby G, Rosenblatt K, Or R, Hodak E (2006). Mycosis fungoides associated with B-cell malignancies. Br J Dermatol.

[60] Wulf GG, Schulz H, Hallermann C, Kunze E, Wörmann B (2001). Reactive polyclonal T-cell lymphocytosis mimicking Sezary syndrome in a patient with hairy cell leukemia. Haematologica..

[61] Prakash A, Khalafallah AA (2018). Concurrent hairy cell leukemia and metastatic merkel cell carcinoma. Case Rep Oncol Med.

[62] Jennette JC, Falk RJ, Bacon PA, Basu N, Cid MC, Ferrario F, Flores-Suarez LF, Gross WL, Guillevin L, Hagen EC, Hoffman GS, Jayne DR, Kallenberg CG, Lamprecht P, Langford CA, Luqmani RA, Mahr AD, Matteson EL, Merkel PA, Ozen S, Pusey CD, Rasmussen N, Rees AJ, Scott DG, Specks U, Stone JH, Takahashi K, Watts RA (2013). 2012 revised International Chapel Hill Consensus Conference Nomenclature of Vasculitides. Arthritis Rheum.

[63] Wooten MD, Jasin HE (1996). Vasculitis and lymphoproliferative diseases. Semin Arthritis Rheum.

[64] Gulati S, Patel NP, Swierczynski SL (2012). Vasculitides associated with haematological malignancies: a case-based review. BMJ Case Rep.

[65] Fain O, Hamidou M, Cacoub P, Godeau B, Wechsler B, Pariès J, Stirnemann J, Morin AS, Gatfosse M, Hanslik T, Belmatoug N, Blétry O, Cevallos R, Delevaux I, Fisher E, Hayem G, Kaplan G, Le Hello C, Mouthon L, Larroche C, Lemaire V, Piette AM, Piette JC, Ponge T, Puechal X, Rossert J, Sarrot-Reynauld F, Sicard D, Ziza JM, Kahn MF, Guillevin L (2007). Vasculitides associated with malignancies: analysis of sixty patients. Arthritis Rheum.

[66] Grey MR, Flanagan NG, Kelsey PR (2000). Severe skin rash in two consecutive patients treated with 2-chlorodeoxyadenosine for hairy cell leukaemia at a single institution. Clin Lab Haematol.

[67] Broccoli A, Gandolfi L, Pellegrini C, Agostinelli C, Argnani L, Zinzani PL (2016) Leukocytoclastic vasculitis associated with hairy cell leukemia at diagnosis: a case report and review of the literature. Tumori 102(Suppl. 2). 10.5301/tj.5000487 10.5301/tj.5000487 27002952

[68] Gabriel SE, Conn DL, Phyliky RL, Scott RE (1986). Vasculitis in hairy cell leukaemia: review of literature and consideration of possible pathogenic mechanisms. J Rheumatol.

[69] Hasler P, Kistler H, Gerber H (1995). Vasculitides in hairy cell leukemia. Semin Arthritis Rheum.

[70] Remkova A, Halcín A, Stenová E, Babál P, Kasperová V, Vranovský A (2007). Acute vasculitis as a first manifestation of hairy cell leukemia. Eur J Intern Med.

[71] Audemard A, Crochette R, Salaün V, Comoz F, Ficheux M (2014). IgA vasculitis revealing hairy cell leukemia relapse treated by cladribine. Presse Med.

[72] Moyers JT, Liu LW, Ossowski S, Goddard L, Kamal MO, Cao H (2019). A rash in a hairy situation: leukocytoclastic vasculitis at presentation of hairy cell leukemia. Am J Hematol.

[73] Farcet JP, Weschsler J, Wirquin V, Divine M, Reyes F (1987). Vasculitis in hairy cell leukemia. Arch Intern Med.

[74] Westbrook CA, Golde DW (1985). Autoimmune disease in hairy cell leukaemia: clinical syndromes and treatment. Br J Haematol.

[75] Tousi B, D’Silva R, Papish S (2002). Systemic vasculitis complicating hairy cell leukaemia treatment with cladribine. Clin Lab Haematol.

[76] Seshadri P, Hadges S, Cropper T (2000). Acute necrotising vasculitis in hairy cell leukemia—rapid response to cladribine: case report and a brief review of the literature. Leuk Res.

[77] International Study Group for Behçet’s disease (1990). Criteria for diagnosis of Behçet’s disease. Lancet.

[78] Oo TH, Delafuente M, Hassoun H (2003). Possible association between hairy cell leukemia and Behçet’s disease. South Med J.

[79] Oksuz MF, Coskun BN, Tufan AN, Orucoglu N, Dalkilic E, Oztürk Nazlıoğlu H, Pehlivan Y (2014). Hairy cell leukemia presenting initially with symptoms of Behçet’s disease. Int J Rheum Dis.

[80] Weinstein AJ (1982). Systemic vasculitis and hairy cell leukemia. J Rheumatol.

[81] Carpenter MT, West SG (1994). Polyarteritis nodosa in hairy cell leukemia: treatment with interferon-alpha. J Rheumatol.

[82] Ozkok A, Elcioglu OC, Akpinar TS, Nalcaci M (2011). Vasculitis in a patient with hairy cell leukemia. Intern Med.

[83] Vankalakunti M, Joshi K, Jain S, Nada R, Radotra BD, Varma S (2007). Polyarteritis nodosa in hairy cell leukaemia: an autopsy report. J Clin Pathol.

[84] Komadina KH, Houk RW (1989). Polyarteritis nodosa presenting as recurrent pneumonia following splenectomy for hairy-cell leukemia. Semin Arthritis Rheum.

[85] Heath MS, Ortega-Loayza A (2019). Insights into the pathogenesis of Sweet’s syndrome. Front Immunol.

[86] Ventura F, Rocha J, Pereira T, Marques H, Pardal F, Brito C (2009). Sweet syndrome as the presenting symptom of hairy cell leukemia. Dermatol Online J.

[87] Levy RM, Junkins-Hopkins JM, Turchi JJ, James WD (2002). Sweet syndrome as the presenting symptom of relapsed hairy cell leukemia. Arch Dermatol.

[88] Dalrì P, Boi S, Cristofolini M, Piscioli F, Rubertelli M (1982). Sweet syndrome: presenting symptom of hairy cell leukemia with fatal infection by Pneumocystis carinii. Haematologica..

[89] Alkayem M, Cheng W (2014). A case report of hairy cell leukemia presenting concomitantly with sweet syndrome. Case Rep Med.

[90] Gisser SD (1983). Acute febrile neutrophilic dermatosis (Sweet’s syndrome) in a patient with hairy-cell leukemia. Am J Dermatopathol.

[91] Shah PR, Scott G, Beck LA (2019). Image gallery: hairy-cell leukaemia presenting with Sweet syndrome. Br J Dermatol.

[92] Özdoğu H, Yeral M, Boğa C (2017). An unusual giant leg ulcer as a rare presentation of Sweet’s syndrome in a patient with hairy cell leukemia successfully managed by splenectomy. Turk J Haematol.

[93] Kromer C, Schön MP, Buhl T (2020). Sweet’s syndrome as precursor of hairy cell leukemia. Dtsch Arztebl Int.

[94] George C, Deroide F, Rustin M (2019). (1019) Pyoderma gangrenosum - a guide to diagnosis and management. Clin Med (Lond).

[95] Janowska A, Oranges T, Fissi A, Davini G, Romanelli M, Dini V (2020). PG-TIME: a practical approach to the clinical management of pyoderma gangrenosum. Dermatol Ther.

[96] Kaplan RP, Newman G, Saperia D (1987). Pyoderma gangrenosum and hairy cell leukemia. J Dermatol Surg Oncol.

[97] Jain A, Lad D, Prakash G, Khadwal A, Malhotra P, Bal A, Mallik N, Kumar N, Varma S (2017). Bullous pyoderma gangrenosum associated with hairy cell leukemia and its complete response to cladribine therapy. Indian J Hematol Blood Transfus.

[98] Tombak A, Aygun S, Serinsoz E, Tiftik EN (2015). Complete recovery of pyoderma gangrenosum after successful treatment of underlying hairy cell leukemia with cladribine. Korean J Intern Med.

[99] Svecová D, Pallová A, Chmurová N, Babal P (2018). Paraneoplastic vasculitis associated with hairy cell leukemia. Prague Med Rep.

[100] Finan MC, Su WP, Li CY (1984). Cutaneous findings in HCL. J Am Acad Dermatol.

[101] Filho RJ, de Carvalho PN, Neto AM, Teixeira Henderson MN, Pinheiro RF (2011). Nontuberculous mycobacterium genital infection mimicking donovanosis in a patient with hairy cell leukemia. Leuk Res.

[102] Weinstein RA, Golomb HM, Grumet G, Gelmann E, Schechter GP (1981). Hairy cell leukemia: association with disseminated atypical mycobacterial infection. Cancer.

[103] Trizna Z, Tschen J, Natelson EA (2001). Atypical mycobacterial infection in a patient with hairy cell leukemia. Cutis..

[104] Castor B, Juhlin I, Henriques B (1994). Septic cutaneous lesions caused by Mycobacterium malmoense in a patient with hairy cell leukemia. Eur J Clin Microbiol Infect Dis.

[105] Maurice PD, Bunker C, Giles F, Goldstone A, Holton J (1988). Mycobacterium avium-intracellular infection associated with hairy-cell leukemia. Arch Dermatol.

[106] Kumar S, Kumar D, Gourley WK, Alperin JB (1994). Sporotrichosis as a presenting manifestation of hairy cell leukemia. Am J Hematol.

[107] Salata RA, King RE, Gose F, Pearson RD (1986). Listeria monocytogenes cerebritis, bacteremia, and cutaneous lesions complicating hairy cell leukemia. Am J Med.

[108] Bettens S, Delaere B, Glupczynski Y, Schoevaerdts D, Swine C (2008). Ecthyma gangrenosum in a non-neutropaenic, elderly patient: case report and review of the literature. Acta Clin Belg.

[109] Sluga R, Tersmette M, Sohne M (2019). Hairy cell leukemia presenting with Ecthyma Gangrenosum- a case report. BMC Infect Dis.

[110] Ganzel C, Gatt ME, Maly A, Ben-Yehuda D, Goldschmidt N (2012). High incidence of skin rash in patients with hairy cell leukemia treated with cladribine. Leuk Lymphoma.

[111] Meher-Homji Z, Tam CS, Siderov J, Seymour JF, Holmes NE, Chua KYL, Phillips EJ, Slavin MA, Trubiano JA (2019). High prevalence of antibiotic allergies in cladribine-treated patients with hairy cell leukemia - lessons for immunopathogenesis and prescribing. Leuk Lymphoma.

[112] Cohen PR, Kurzrock R (1999). 2-chloro-deoxyadenosine-associated transient acantholytic dermatosis in hairy cell leukemia patients. Am J Dermatopathol.

[113] Chubar Y, Bennett M (2003). Cutaneous reactions in hairy cell leukaemia treated with 2-chlorodeoxyadenosine and allopurinol. Br J Haematol.

[114] Espinosa Lara P, Quirós Redondo V, Aguado Lobo M, Jiménez-Reyes J (2017). Purpuric exanthema in a patient with hairy cell leukemia treated with cladribine and allopurinol. Ann Hematol.

[115] Castagna J, Amsler E, Kurihara F, Chasset F, Barbaud A, Soria A (2020). Atypical features of cutaneous adverse drug reactions during therapy for hairy cell leukemia. J Allergy Clin Immunol Pract.

[116] Zevin S, Hershko C, Rosenmann E (1996). Halogenoderma of the forearm caused by 2-chlorodeoxyadenosine treatment. Am J Hematol.

[117] Li C, Geng H, Ji L, Jiang Y, Ma X, Yin Q, Xiong H (2019). Severe polymorphic erythema due to interferon α-2b during treatment of hairy cell leukemia. J Int Med Res.

[118] Urosevic-Maiwald M, Nobbe S, Kerl K, Benz R (2014). Disseminated ulcerating lupus panniculitis emerging under interferon therapy of hairy cell leukemia: treatment- or disease-related?. J Dermatol.

[119] Carlos G, Anforth R, Clements A, Menzies AM, Carlino MS, Chou S, Fernandez-Peñas P (2015). Cutaneous toxic effects of BRAF inhibitors alone and in combination with MEK inhibitors for metastatic melanoma. JAMA Dermatol.

[120] Tiacci E, De Carolis L, Simonetti E, Zaja F, Capponi M, Ambrosetti A, Lucia E, Antolino A, Pulsoni A, Ferrari S, Zinzani P, Rigacci L, Gaidano G, Della Seta R, Frattarelli N, Falcucci P, Visani G, Foa R, Falini B (2019). The BRAF inhibitor vemurafenib plus rituximab produces a high rate of deep and durable responses in relapsed/refractory hairy cell leukemia: updated results of a phase-2 trial. Hematol Oncol.

[121] Bellinato F, Maurelli M, Gisondi P, Girolomoni G (2020). Clinical features and treatments of transient acantholytic dermatosis (Grover’s disease): a systematic review. J Dtsch Dermatol Ges.

[122] Singh AG, Tchanque-Fossuo CN, Elwood H, Durkin JR (2020). BRAF inhibitor and hairy cell leukemia-related transient acantholytic dermatosis. Dermatol Online J.

